# Introgression of Powdery Mildew Resistance Gene *Pm56* on Rye Chromosome Arm 6RS Into Wheat

**DOI:** 10.3389/fpls.2018.01040

**Published:** 2018-07-17

**Authors:** Ming Hao, Meng Liu, Jiangtao Luo, Chaolan Fan, Yingjin Yi, Lianquan Zhang, Zhongwei Yuan, Shunzong Ning, Youliang Zheng, Dengcai Liu

**Affiliations:** ^1^Triticeae Research Institute, Sichuan Agricultural University, Ya’an, China; ^2^Crop Research Institute, Sichuan Academy of Agricultural Science, Chengdu, China

**Keywords:** *Blumeria graminis*, cereal rye, powdery mildew, *Secale cereale*, *Triticum aestivum*

## Abstract

Powdery mildew, caused by the fungus *Blumeria graminis* f. sp. *tritici*, represents a yield constraint in many parts of the world. Here, the introduction of a resistance gene carried by the cereal rye cv. Qinling chromosome 6R was transferred into wheat in the form of spontaneous balanced translocation induced in plants doubly monosomic for chromosomes 6R and 6A. The translocation, along with other structural variants, was detected using *in situ* hybridization and genetic markers. The differential disease response of plants harboring various fragments of 6R indicated that a powdery mildew resistance gene(s) was present on both arms of rye chromosome 6R. Based on karyotyping, the short arm gene, designated *Pm56*, was mapped to the subtelomere region of the arm. The Robertsonian translocation 6AL⋅6RS can be exploited by wheat breeders as a novel resistance resource.

## Introduction

*Blumeria graminis* f. sp. *tritici* (Bgt), the causative fungus of powdery mildew disease, can be a highly destructive pathogen of wheat. The disease is effectively controlled by host genetic resistance, but historically the pathogen has quite rapidly overcome widely deployed host resistance genes. Up to now, more than 70 formally designated powdery mildew resistance genes (*Pm*) have been identified in wheat (McIntosh et al., 2013; Liu et al., 2017; Wiersma et al., 2017). A significant number of these genes are derived from cultivated or wild relative species in the tertiary gene pool; species whose chromosomes do not normally pair with wheat homoeologs; for example, *Thinopyrum intermedium* (*Pm40, Pm43, PmL962*) (He et al., 2009; Luo et al., 2009; Shen et al., 2015), *Th. ponticum* (*Pm51*) (Zhang et al., 2014), *Dasypyrum villosum* (*Pm21* and *Pm55*) (Chen et al., 1995; Zhang et al., 2016), *Agropyron cristatum* (a gene on chromosome 2P, as yet unnamed) (Li H. et al., 2016), and cereal rye (*Secale cereale*) (*Pm7, Pm8/Pm17*, and *Pm20*) (Heun and Friebe, 1990; Friebe et al., 1994; Hsam and Zeller, 1997; Ren et al., 2009). Of the rye-derived genes, only *Pm20* is currently still effective in China (Zhou et al., 2002).

Balanced translocations, in which a wheat chromosome arm is fused to the opposite arm of a homoeologous chromosome present in a donor species belonging to the tertiary gene pool, represent a key intermediate in the process of alien introgression. Such so-called Robertsonian translocations are rather frequently induced during meiosis via the breakage-fusion of a pair of non-paired monosomes (Lukaszewski and Gustafson, 1983; Lukaszewski, 1997; Friebe et al., 2005). One of the best known of these in wheat is the 1BL⋅1RS translocation (Mettin et al., 1973; Zeller, 1973), which was used widely until recent times. A 6AL⋅6VS translocation carrying *Pm21* is present in some Chinese cultivars (Chen et al., 1995; Cao et al., 2011).

The Chinese cereal rye cultivar Qinling (QL) is immune to powdery mildew. Here, as a means of introgressing its resistance into wheat, a disomic 6R(6A) substitution line was developed, which proved to be immune to powdery mildew. To induce Robertsonian translocations, double monosomic (6A + 6R) individuals were produced by crossing the substitution line with euploid, and these plants were allowed to self-pollinate. A number of cytogenetic and genotypic assays were applied to demonstrate the induction of breakage-fusion events, leading to the recognition that the 6R chromosome harbored *Pm* genes on both of its arms. Plants carrying a 6AL⋅6RS Robertsonian translocation proved to be immune to powdery mildew.

## Materials and Methods

### Plant Materials

The wheat lines used comprised cv. Kaixian-luohanmai (KL), the breeding lines D-2-3-4 and SY95-71, and the Chinese spring (CS) *ph1b* mutant (Sears, 1977), while the cereal rye cultivar used was cv. QL. D-2-3-4 is a selection from the cross KL × CS, and was used because its F_1_ hybrids with rye were known to be partially fertile (our unpublished data). SY95-71 is a highly powdery mildew susceptible breeder’s line used as a disease spreader. D-2-3-4 was crossed as female with cv. QL, and a single F_2_ progeny was advanced to the F_3_ generation. A number of the resulting F_3_ plants were repeatedly selfed to F_11_, at which point the material was analyzed both for karyotype and reaction to powdery mildew infection in the field. One of the powdery mildew resistant selections (female), which proved to be a disomic substitution of 6A by 6R [6R(6A)], was crossed to both KL (male) and CS*ph1b* (male) to generate double monosomic (6A + 6R) plants, the selfed progeny of which were assessed for powdery mildew reaction in the greenhouse.

### Molecular Marker Analysis

Genomic DNA was isolated from seedling leaves using a Plant Genomic DNA kit (Tiangen, Beijing, China) and screened with the PCR-based markers KU.825, KU.962, KU.496, and KU.824; the former two markers detect loci on 6RS proximal and distal regions, respectively, and the latter two loci on 6RL proximal and unknown regions, respectively (Qiu et al., 2016, **Table 1**). The chromosome-based draft genome sequence of CS (International Wheat Genome Sequencing Consortium [IWGSC], 2014) was used to develop markers specific to both 6AS and 6AL (Zeng et al., 2016): the former (6AS-LM) was based on the *Traes_6AS_FA2CBD782* and the latter (6AL-LM) on the *Traes_6AL_FEF586A96* sequence (**Table 1**). Each 20 μl PCR comprised 8 μl ddH_2_O, 10 μl 2× Taq PCR MasterMix (Biomed, Beijing, China), 0.5 μl 10 μM forward primer, 0.5 μl 10 μM reverse primer, and 1 μl of ∼100 ng/μl DNA template. The reactions were subjected to a denaturation of 94°C/5 min, followed by 35 cycles of 95°C/30 s, 58°C/30 s, 72°C/90 s, and were completed by an extension of 72°C/10 min. The resulting amplicons were separated by 1.5% agarose gel electrophoresis and visualized by EtBr staining.

**Table 1:** PCR markers used to score for the presence/absence of chromosomes 6A and 6R.

Marker	Forward primer sequence	Reverse primer sequence	Size (bp)	Tm (°C)
6AS-LM	ACTCCTTTCACCATTCCATCCTT	ACTGAAATGAGATGAGCTCACAG	640	58
6AL-LM	GCAAAAGGATAAGATGTATCGTC	CCTCTAGATGATCAGACTCCTTG	840	58
KU.825	GGTCATCAATGACTTGCGTGT	CCTGATGTATGCCCCAAAAA	400	60
KU.962	GGACTTCCTTGTGGCTCAGG	TGTCAGGGCACCAGTGATAA	400	60
KU.496	CTCGCCCTGGTATCACTTTC	TCCTCGCTCCTAAAACATGC	400	60
KU.824	CGGTTAGCTTTAGCCACGAC	GCACGTGAATGAAATCGTTG	400	60

### Cytogenetic Assays

Genomic *in situ* hybridization (GISH) was performed as described by Hao et al. (2011). The hybridization mixture was composed of 7.5 μl 100% formamide, 1.5 μl 20× SSC, 1 μl ∼75 ng/μl labeled QL genomic DNA, 2 μl ∼4.5 μg/μl sheared CS genomic DNA, 1.5 μl 10 mg/ml sheared salmon sperm DNA, and 3 μl 5% w/v dextran sulfate. Fluorescence *in situ* hybridization (FISH) was performed as described by Komuro et al. (2013). The FISH probes used were oligo-pSc119.2 (Rayburn and Gill, 1985), (GAA)_5_ (Pedersen et al., 1996), oligo-pTa535, oligo-pTa713 (Zhao et al., 2016), oligo-CCS1 (centromere probe) (Tang et al., 2014), and telomere probe (TTTAGGG)_3_ (Werner et al., 1992). Oligo-pSc119.2 preferentially paints tandem repeats on B- and R-genome chromosomes. (GAA)_5_ preferentially paints tandem repeats on A- and B-genome chromosomes. Oligo-pTa535 preferentially paints tandem repeats on D- and A-genome chromosome. Oligo-pTa713 results in distinctive bands on multiple chromosome arms and especially useful in identifying individual chromosome arms of wheat. The probes were synthesized and either FAM- or TAMRA-labeled by TsingKe Biological Technology Company (Chengdu, China).

### Assessment of Reaction to Powdery Mildew Infection

Powdery mildew reactions were observed in field-grown plants raised at Sichuan Agricultural University’s Ya’an (2010–2011) and Wenjiang (2010–2014) Experimental Stations, where natural infection is common (Liu et al., 2015). Each genotype was represented by three replicates of 20 plants grown in 2 m long rows separated from one another by 30 cm. The highly susceptible common wheat line SY95-71 was planted on both sides of each experimental row and used to increase Bgt inoculum and as the susceptible control. The host reaction was evaluated at the heading and grain filling stages by scoring on a 0–4 scale following Si et al. (1992). Powdery mildew reactions of progenies from double monosomic plants were determined in a small (15 m^2^ in area) greenhouse in order to a well-controlled condition. Greenhouse-based disease reactions were determined by inoculating seedlings at the three leaf stage of progenies and susceptible wheat parents held under a 16 h photoperiod, 22°C (light)/16°C (dark) regime at a relative humidity of 75%; the inoculum was a mixture of local Bgt conidiospores, which were dusted onto the leaves. Plants were inspected at 15-day intervals, and were scored using the Si et al. (1992) scale.

## Results

### Substitution and Addition Lines Resistant to Powdery Mildew

Of 10 D-2-3-4 × QL F_1_ hybrids, one produced three F_2_ progeny, and of these, only one was self-fertile. After selfing this individual through to the F_11_ generation with selection for good fertility, 42 lines were selected for karyotyping using GISH and FISH. Ten of these lines were found to contain rye chromatin: nine were 6R(6A) disomic substitutions and one was a disomic 2R addition line. All the 10 lines with rye chromatin were immune to powdery mildew in the field over the 2010–2014 seasons, whereas the other 32 lines were fully susceptible.

### Detecting 6R Structural Variants in the Progeny of the 6A + 6R Double Monosomic Plants

**FIGURE 1:**
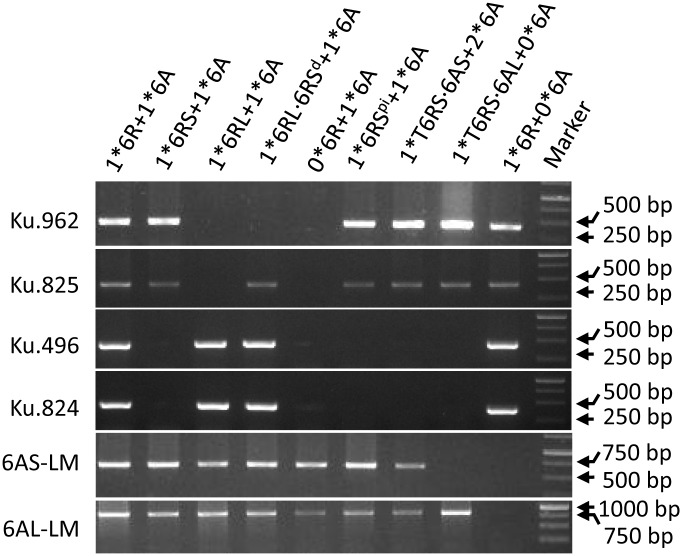
PCR profiling of (from left to right) LM51, LM52, LM61, HM99, LM57, HM109, HM148, LM47, and HM100, using assays for loci on chromosomes 6A and 6R.

**FIGURE 2:**
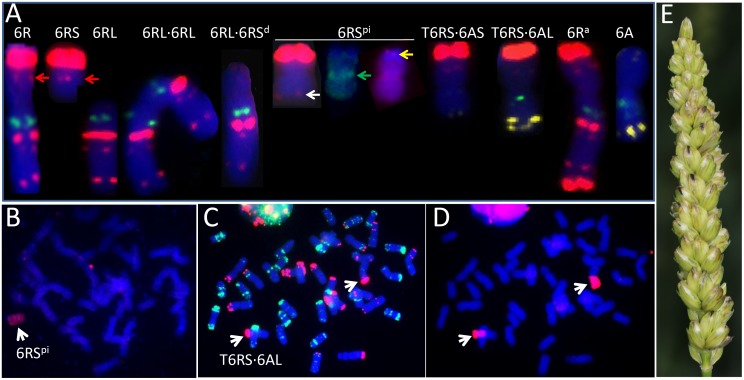
Structural variations of chromosome 6R recovered among the progeny of 6A + 6R double monosomic plants. **(A)** Variants recovered in the first selfing generation. pSc119.2 sites and the telomere [(TTTAGGG)_3_] (6RS^pi^, yellow arrow) are shown in red, pTa713 sites in yellow, (GAA)_5_ and CCS1 (6RS^pi^, green arrow) sites in green. The red arrow shows the pSc119.2 site in the center of a normal 6RS arm and the white arrow a minor pSc119.2 site at the terminus of the 6R paracentric inversion (6RS^pi^). **(B)** GISH karyotype of HM109, carrying the 6R paracentric inversion (6RS^pi^). Rye chromatin is colored red. Sequential FISH **(C)** and GISH **(D)** karyotype of LM47-6, which carries a pair of 6AL⋅6RS translocations. The red and green colored sites, respectively, are pSc119.2 and pTa535 sites **(C)**, while the rye chromatin is colored red **(D)**. **(E)** A spike of homozygous T6RS⋅6AL translocation line. Pi indicates paracentric inversion.

The DS6R(6A) line was crossed with KL and CS*ph1b* to generate double monosomic plants. The 6A + 6R double monosomic plants were fully self-fertile. In all, 69 progenies were characterized genotypically and karyotypically (Supplementary Table S1); of these, 25 were descended from three 6R(6A) × CS*ph1b* hybrids (HM922-4, -17, and -19) and 44 from four 6R(6A) × KL hybrids (HM923-2, -4, -5, and -9). Ten progenies [two from the 6R(6A) × CS*ph1b* cross and eight from the 6R(6A) × KL cross] lacked both the 6AS and 6AL markers, and hence were deemed to lack the entire chromosome; similarly, 18 [5 from the 6R(6A) × CS*ph1b* cross and 13 from the 6R(6A) × KL cross] lacked the markers for both arms of 6R, so were deemed to have lost the entire 6R (**Figure 1**). Among the 6R(6A) × CS*ph1b* progeny, 14 were monosomic for an intact copy of 6R and 2 were disomic, while 5 harbored either a misdivided or a translocated version of 6R (**Table 2**); the equivalent numbers for the 6R(6A) × KL progeny were 18 6R monosomics, 6 6R disomics, and 9 harboring a misdivided or a translocated version of 6R. The 14 misdivided or translocated versions were classified into the following six types (**Figure 2A** and **Table 2**):

(1) A telosome, either 6RS (LM52, LM74, HM151) or 6RL (LM61, LM98).
(2) An isochromosome for 6RL (HM136).
(3) A near entire chromosome 6RL⋅6RS^d^ lacking the telomeric end of 6RS (LM99 and HM99).
(4) A chromosome 6RS^pi^ possibly carrying an paracentric inversion (HM109, **Figure 2B**). All two 6RS markers had been retained (**Figure 1**), but the two pSc119.2 sites in the center of a normal 6RS (**Figure 2A**, red arrow) appeared as two weaker sites near the telomere (**Figure 2A**, white arrow). FISH results using the centromere probe indicated that it contained a centromere in the middle (**Figure 2A**, green arrow). FISH based on the telomere sequence indicated that a full telomere was only present on the arm carrying the major pSc119.2 terminal site (**Figure 2A**, yellow arrow).
(5) The Robertsonian translocations 6AL⋅6RS (LM47), 6AS⋅6RS (HM148, HM156), and 4AL⋅6RS (LM30).
(6) A form of 6R (6R^a^) carrying two additional major pSc119.2 sites toward the end of 6RL (LM43).

**Table 2:** First generation selfed progeny of 6A + 6R double monosomic plants carrying variant forms of 6R and their response to powdery mildew infection.

Hybrid combinations	Code	PCR marker analysis						Constitution of 6A and 6R by FISH and/or GISH analysis	Infection types
		6AS-LM	6AL-LM	6RS-Ku.962	6RS-Ku.825	6RL-Ku.496	6RL-Ku.824		
DS6R(6A)/CS*ph1b*	HM136	−	−	−	−	+	+	0^∗^6A + 1^∗^6RL⋅6RL	1−2
	LM47	−	+	+	+	−	−	0^∗^6A + 1^∗^T6RS⋅6AL	0
	LM52	+	+	+	+	−	−	1^∗^6A + 1^∗^6RS	0
	LM43	+	+	+	+	+	+	1^∗^6A + 1^∗^6R^a^	0
	LM30	+	+	+	+	+	+	2^∗^6A + 1^∗^6R + 1^∗^T6RS⋅4AL	0
DS6R(6A)/KL	LM61	+	+	−	−	+	+	1^∗^6A + 1^∗^6RL	1−2
	LM99	+	+	−	+	+	+	1^∗^6A + 1^∗^6RL⋅6RS^d^	1−2
	HM99	+	+	−	+	+	+	1^∗^6A + 1^∗^6RL⋅6RS^d^	1−2
	LM74	+	+	+	+	−	−	1^∗^6A + 1^∗^6RS	0
	HM151	+	+	+	+	−	−	1^∗^6A + 1^∗^6RS	0
	HM109	+	+	+	+	−	−	1^∗^6A + 1^∗^6RS^pi^	0
	HM148	+	+	+	+	−	−	2^∗^6A + 1^∗^T6RS⋅6AS	0
	LM98	+	+	+	+	+	+	1^∗^6A + 1^∗^6R + 1^∗^6RL	0
	HM156	+	+	+	+	+	+	1^∗^6A + 1^∗^6R + 1^∗^T6RS⋅6AS	0

*^∗^Was used to space the number of chromosome and the name of chromosome.*

### Transmission of the Modified Forms of 6R and the Occurrence of *de Novo* Modified Forms

The transmission of the modified forms of 6R identified in the progeny of the first selfing generation of the 6A + 6R double monosomic was quantified by an analysis of the derived second generation families (**Table 3**). The 6AS⋅6RS translocation present in HM148 was retained by 6 out of 13 individuals, one of which was disomic for the translocation. The near entire chromosome 6R present in HM99 was transmitted to two out of seven progeny, and the same transmission rate was observed for the 6RS telosome present in HM151. The rate of transmission of the paracentric inversion present in HM109 was only one out of eight. The 6AL⋅6RS translocation in LM47 was present in 9 out of 11 progeny, one of which carried two copies of the translocation (**Figures 2C–E**). Four further structural variants were identified in the second generation (**Table 3**). The first was a 6RL telosome found in one individual derived from HM99 (which carried the truncated form of 6R); the second was a truncated form of 6R lacking a terminal part of the short arm, derived from HM103 (a plant carrying a 6R monosome); the third was a 6RS isochromosome derived from LM47 (which harbored 6AL⋅6RS); and the fourth, a 6AL⋅6RS translocation detected among the progeny of HM166, the karyotype of which had not been determined.

**Table 3:** The chromosome 6R constitution of the second selfing generation of 6A/6R double monosomic plants and their responses to powdery mildew infection.

Hybrid combinations	Code	Constitution of 6A and 6R by FISH and/or GISH analysis	Infection types
DS6R(6A)/KL	HM99-3, -5, -9	1^∗^6A + 0^∗^6R	4
	HM99-6	2^∗^6A + 0^∗^6R	4
	HM99-1	1^∗^6A + 1^∗^6RL	1−2
	HM99-2, -8	1^∗^6A + 1^∗^6RL⋅6RS^d^	1−2
	HM109-3, -4, -6, -7, -9	1^∗^6A + 0^∗^6R	4
	HM109-1, -5	2^∗^6A + 0^∗^6R	4
	HM109-2	1^∗^6A + 1^∗^6RS^pi^	0
	HM151-1, -3, -4, -7	1^∗^6A + 0^∗^6R	4
	HM151-5	2^∗^6A + 0^∗^6R	4
	HM151-2, -6	1^∗^6A + 1^∗^6RS	0
	HM148-14	1^∗^6A + 0^∗^6R	4
	HM148-1, -2, -4, -8, -10, -15	2^∗^6A + 0^∗^6R	4
	HM148-3, -7, -9, -12, -13	2^∗^6A + 1^∗^T6RS⋅6AS	0
	HM148-5	2^∗^6A + 2^∗^T6RS⋅6AS	0
	HM103-2	0^∗^6A + 1^∗^6RL⋅6RS^d^	1−2
DS6R(6A)/CS*ph1b*	LM47-7, -10	0^∗^6A + 0^∗^6R	4
	LM47-1, -2, -4, -5, -8, -11, -12	0^∗^6A + 1^∗^T6RS⋅6AL	0
	LM47-6	0^∗^6A + 2^∗^T6RS/6AL	0
	LM47-9	1^∗^T6RS⋅6AL + 1^∗^6RS⋅6RS	0
	HM166-1	1^∗^6AS + 1^∗^T6RS⋅6AL	0

*^∗^Was used to space the number of chromosome and the name of chromosome.*

### Both Arms of Chromosome 6R Harbor Genes Conditioning Resistance Against Powdery Mildew

**FIGURE 3:**
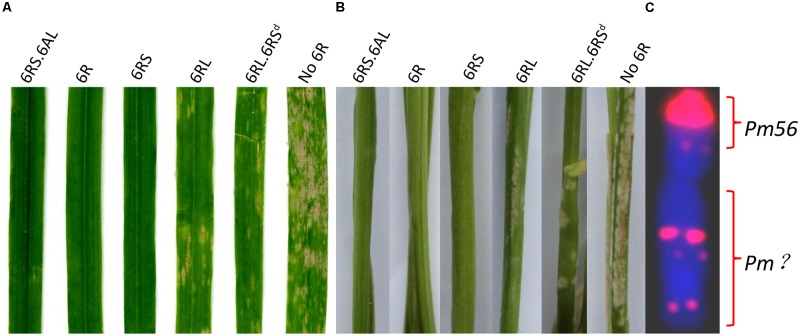
The response to powdery mildew infection on **(A)** the leaf and **(B)** the leaf sheath of plants containing various forms of chromosome 6R. **(C)** The location of *Pm* genes on cv. QL chromosome 6R. Left to right: carriers of T6AS⋅6RS, 6R, 6RS, 6RL, a truncated form of 6R lacking a subtelomeric region of 6RS (6RL⋅6RS^d^), and a plant lacking 6R (No 6R). The red signals on the 6R chromosome in **(C)** represent pSc119.2 sites.

All of the first selfing generation plants harboring an entire 6R proved to be immune to powdery mildew when tested in the greenhouse, whereas all those without 6R chromatin were highly susceptible (Supplementary Table S1), as were the nine second generation plants lacking 6R (**Table 3**). This outcome was consistent with the field assessment, confirming that powdery mildew resistance was conferred by gene(s) mapping to chromosome 6R. When the disease response of plants derived from the 6R(6A) × CS*ph1b* cross harboring incomplete forms of 6R (**Figure 3** and **Tables 2, 3**) were compared, it was clear that the presence of the short arm was associated with immunity, while the presence of the long arm alone did not confer immunity, but rather only a high level of resistance. The implication was that both arms harbor a resistance gene(s), with the one(s) on the short arm being more effective than the one(s) on the long arm. A consistent conclusion was drawn from the equivalent analysis of the progeny of the 6R(6A) × KL cross: all plants which had retained the entire 6RS arm were immune, while those which retained 6RL but lacked the terminal end of 6RS were moderately to highly resistant. Since the truncated form of 6R (LM99, HM99, HM99-2, HM99-8, and HM103-2) lacked the 6RS subtelomeric region, the assumption was that the gene conferring immunity to powdery mildew lies in the truncated segment (**Figure 3C**). The PCR marker KU.962 specific for 6RS also failed to amplify in plants with 6RL⋅6RS^d^ (**Figure 1**), indicating that the sequence is also located in this chromosome region and that this PCR-based marker will be useful in further investigation or chromosome engineering of the resistance gene. However, it is unclear for the genetic distance between marker KU.962 and the resistance gene.

## Discussion

Powdery mildew is a damaging disease of the world wheat crops, especially in Sichuan Province, China, where it can cause very high yield losses (Liu et al., 2012). The race structure of Bgt in Sichuan is complex, with as many as 109 distinct isolates having been obtained from a set of 327 infected leaves. Of 28 known *Pm* genes, only *Pm21* was able to mount an immune response to these isolates (Liu et al., 2015).

The present experiments have established that wheat plants carrying chromosome 6R of cereal rye cv. QL were immune to powdery mildew in Sichuan, and that resistance genes were present on both of its arms. The long arm of 6R carried by cv. Prolific is known to harbor *Pm20*, a gene which conditions immunity against a range of Bgt isolates (Heun and Friebe, 1990; Friebe et al., 1994), while Li M. et al. (2016) have mapped a powdery mildew resistance gene to the same arm in cv. Kustro and wheat plants carrying 6RL transferred from the rye germplasm accession PI 252003 manifest an isolate-specific response to infection (Friebe et al., 1994). Here, the presence of chromosome arm 6RS was associated with an immune reaction, although no *Pm* gene has previously been reported to map to this arm. Assuming that this immunity is conditioned by a single gene, the gene can be provisionally designated *Pm56*. Structural variation in the 6RS arm has allowed *Pm56* to be localized to the terminal region of the arm, lying between the telomere and the most distal pSc119.2 site (**Figure 3C**). The 6RS arm is largely syntenic with the wheat homeologous group 6 short arms (Martis et al., 2013), although its terminal segment was translocated to 2R (Devos et al., 1993). Thus, of the two wheat/rye Robertsonian translocations isolated, the expectation is that 6AL⋅6RS will provide a higher level of genetic compensation than 6AS⋅6RS, so is likely to prove more useful for breeding purposes.

Wheat plants in which both a wheat and a non-wheat chromosome are present in the monosomic state have long been known to provide a source of Robertsonian translocations: some recent examples have been provided by Ardalani et al. (2016) and Rahmatov et al. (2016). Here, the use of *in situ* hybridization and genotyping has led to the detection of a number of novel chromosomes involving segments of chromosome 6R; notably, among 69 selfed progeny of a double monosomic for 6A and 6R, 4 carried a Robertsonian translocation, assumed to have been induced by the breakage-fusion of the two unpaired chromosomes (Lukaszewski, 1997; Friebe et al., 2005).

The 6R(6A) substitution arose following the repeated selfing of a partially fertile wheat-rye amphihaploid plant. Although such plants are generally self-sterile, a low level of fertility can be restored through the process of first division restitution (Ramanna and Jacobsen, 2003). The phenomenon has been recorded in synthetic wheat/rye amphihaploids (Zeng et al., 2014): the selfed progeny of these crosses include some partial amphiploids (Zeng et al., 2014). Repeated selfing of these individuals allied to selection for fertility favors the production of hexaploids of genomic constitution AABBRR (Hao et al., 2013). However, in both the present case and that described by Yuan et al. (2014), the final products were either euploid bread wheat or a disomic substitution line, reflecting the elimination of all or most of the rye, rather than of the D genome chromosomes. A possible cytological explanation for the low fertility of the amphihaploid and the subsequent loss of the rye complement has been suggested by Silkova et al. (2011), who observed a high frequency of equational and reductional divisions in bread wheat/rye hybrids. If equational division predominated for the wheat chromosomes and reductional division for the rye chromosomes, then gametes carrying an unreduced wheat complement along with a reduced rye one could have formed. The union of such uniparental unreduced gametes can be expected to result in the formation of a partial amphidiploids containing two sets of all or most of the wheat complement, leaving the rye chromosomes in the monosomic state. In subsequent generations, the rye chromosomes would be largely eliminated.

## Data Availability

The seeds harboring homozygous 6AL⋅6RS translocation is available upon request to MH, Triticeae Research Institute, Sichuan Agricultural University, at haomingluo@foxmail.com.

## Author Contributions

MH and DL conceived and designed the experiments. DL, LZ, and MH developed the substitution lines. ML, JL, CF, and YY performed the cytogenetic experiments. ML, CF, and SN molecular marker analysis. ML, JL, LZ, and ZY evaluated the powdery mildew resistance. YZ and DL supervised the study. ML, MH, and DL wrote the paper.

## Conflict of Interest Statement

The authors declare that the research was conducted in the absence of any commercial or financial relationships that could be construed as a potential conflict of interest.
